# COVID-19: Establishing, implementing and assessment safety strategy, descriptive interventional study

**DOI:** 10.1371/journal.pone.0283197

**Published:** 2023-03-17

**Authors:** Tarig M. S. Alnour, Osama Al-Amer, Nizar Hamed Saeedi, Abdullah Shater, Mohamed A. Alsuba, Eltayib H. Ahmed-Abakur

**Affiliations:** 1 Medical Laboratory Technology Department, Faculty of Applied Medical Science, University of Tabuk, Tabuk, Saudi Arabia; 2 Department of Microbiology and Immunology, Faculty of Medical Laboratory Science, Alzaiem Alazhari University, Khartoum, Sudan; Mashhad University of Medical Sciences, ISLAMIC REPUBLIC OF IRAN

## Abstract

The increase in severe acute respiratory syndrome SARS-CoV-2 has invariably affected medical professionals in their training, academic and professional development. The present study was an interventional descriptive study aimed at reducing the risk of exposure to COVID-19 and enabling physical attendance to the practical session for applied medical students by establishing and implementing a safety strategy. The adopted safety strategy has eight conditions and 50 requirements. Compliance with the safety strategy along with the serological diagnosis of COVID-19 was used as a key performance indicator for assessing the efficiency of the safety strategy. A total of 197 students were enrolled at the beginning of the study. The overall results showed that 78.1% of the respondents strictly followed the protocol, 14.5% of the individuals partially responded to the protocol and 7.4% of the individuals did not respond to the protocol. Twenty-two (12.6%) out of the 175 participants who completed the study had positive COVID-19 during the study period, whereas the remaining 153 participants (87.4%) appeared to be healthy. The serological results showed that 68 (38.9%) and 66 (37.7%) individuals of the study population had positive IgM+IgA and IgG of COVID-19, respectively; the majority of the participants who developed antibodies did not show symptoms and appeared to be healthy during the study. The physical distancing condition was the only condition that displayed a significant relationship with seropositive IgM+IgA. The compiling of standardized protocols along with serological diagnoses can be an effective tool in measuring the effectiveness of safety protocol and reducing the risk of exposure to COVID-19.

## Introduction

Coronavirus disease 2019 (COVID-19), which is caused by SARS-CoV-2, is an unprecedented infection that has rapidly spread throughout the world, thus leading the WHO to declare it a global public health emergency on January 30, 2020 [1–3]. However, during the last two decades, several epidemics have occurred and are associated with viruses, including Middle East Respiratory Syndrome (MERS), H1N1 influenza and Severe Acute Respiratory Syndrome (SARS-COV). The new SARS-CoV-2 virus appears to be very contagious and more dangerous than previous viruses [4]. It has caused 6,149,207 million deaths out of 482,330,087 million confirmed and stated cases worldwide as of March 28, 2022 [5]. COVID-19 patients show symptoms that vary from mild forms to clinical conditions characterized by fever, pneumonia and respiratory distress syndrome [6–8]. Moreover, the virus is easily transmitted, and infection can occur by direct contact with the infected person and indirect contact with surfaces in the immediate environment [8–10].

The present situation of a highly contagious pandemic disease, the unavailability of effective treatment and broad methods of transmission have led to the suspension of many routine activities throughout the world. The education system, as well as other fields, have been significantly affected by this pandemic disease. The effect on medical education has a special concern, as it may have negative subsequent effects on the future of the medical field and the wellbeing of the community [11]. One of the solutions for this dilemma involves the online learning system. During the last outbreak of H5N1, several universities around the world have utilized this system as an alternative teaching system [12]. It appears to be a suitable option to compensate for the lack of regular study and to overcome the closing. Nevertheless, practical classes have emerged as a real challenge, particularly in applied medical colleges.

In Saudi Arabia, the first incident of COVID-19 was detected on March 2, 2020, after which the number of cases continually increased at a rate of fifty-two cases per day. One month later, the number of cases greatly increased at an average rate of 750 cases per day. This trend of increasing cases has grown and reached its peak in June, with an average rate of 3,570 cases per day. The total number of reported COVID-19 cases in Saudi Arabia represents 0.7% of the population [13], with a death rate of approximately 1–2% [14].

To address this situation and the possible emergence of similar infectious diseases, as well as to empower the online learning system, the University of Tabuk, Saudi Arabia, derived a safety strategy from the Center for Disease Control and Prevention (CDCP) and from the guidelines of the local authorized bodies “Ministry of Health and Ministry of Education” [15–19],which was aimed at lowering the risk of exposure to COVID-19 infection and to allow for the physical attendance of practical sessions. The present study aimed to assess the efficiency of the designated safety strategy. Compliance with the safety strategy for infectious disease (COVID-19) was assessed along with serological diagnoses, and they were used as key performance indicators for evaluating the efficiency of the safety policy. This study was performed before the implementation of the COVID-19 vaccine.

## Materials and methods

The present study was an interventional descriptive study; involved the establishment and implementation of a safety strategy against COVID-19 to reduce the risk of exposure to COVI-19 and to allow for normal activities during the suspension period. The established strategy comprised eight conditions involving 50 requirements. It was conducted over 16 working weeks (during one academic semester) from September 1, 2020, to December 31, 2020.

The ethical clearance of this study (UT-135-08-2021) was obtained from the research ethics committee of the University of Tabuk, Saudi Arabia. The participants were informed about the purposes of the study, and signed consent were obtained from them.

### Study population

Undergraduate students of applied medical sciences at the University of Tabuk who had physically attended (face to face) practical classes represented the study population. The study population was divided into two main groups: the infected group and the healthy group. The infected group included the participants who had positive PCR results for COVID-19 and showed symptoms during the study period, whereas the healthy group included the participants who neither had positive PCR for COVID-19 nor showed symptoms. The healthy group was further subdivided into a mixed group involving those individuals who had close contact with COVID-19 patients, and the non-mixed group included those individuals who did not have contact with COVID-19 patients. At the beginning of the study, all of the participants were lacking in positive PCR data for COVID-19 and symptoms of COVID-19, and they had been trained on practicing the safety strategy before the initiation of the study. Moreover, the participants were motivated and followed up to comply with the strategy.

### Development and assessment of safety strategy against COVID-19

The safety strategy that was developed and implemented comprised eight conditions involving 50 requirements (Table 1): signs, posters and engineering control conditions (five requirements), laboratory procedures upon entry conditions (five requirements), physical distancing conditions (five requirements), hand hygiene and respiratory etiquette conditions (eleven requirements), mandatory disinfection conditions (ten requirements), cleaning and waste disposal conditions (five requirements) and modified suitable laboratory teaching strategies condition (four requirements) and laboratory procedure prior to leaving the room condition (five requirements).

**Table 1:** Response to the safety strategy toward COVID-19.

Conditions	Requirements		Response		
			Yes	No	Some time
**Laboratory Procedure Upon Entry**	Wear mask properly		183/197 (92.9%)	3/197 (1.5%)	11/197 (5.6%)
	Wear laboratory coats properly		162/197 (83.8%)	20/197 10.1%)	15/197 (7.6%)
	Temperature status is assessed daily		127/197 (64.5%)	27/197 (13.7%)	43/197 (21.8%)
	Enter the door individually (2-meter distance)		121/197 (61.4%)	23/197 (11.7%)	53/197 (26.9%)
	Disinfect the designated working area.		129/197 (65.5%)	28/197 (14.2%)	40/197 (20.3%)
Mean			722/985 = 73.3	101/985 = 10.3	162/985 = 16.4
**Physical Distancing**	Stay to designated seats & avoid moving around		135/197 (68.5%)	16/197 (8.1%)	46/197 (23.4%)
	Avoid close contact gestures (Hand shaking, close, greetings)		131/197 (66.5%)	25/197 (12.7%)	41/197 (20.8%)
	Individual and independent work is mandatory (no group)		123/197 (62.4%)	28/197 (14.2%)	46/197 (23.4%)
	Equipment assigned exclusively to single student		115/197 (58.4%)	41/197 (20.8)	41/197 (20.8)
	Maintain 2-meter distance at all times		113/197 (57.4%)	34/197 (17.3%)	50/197 (25.4%)
Mean			617/985 = 62.6	144/985 = 14.6	224/985 = 22.7
**Hand Hygiene And Respiratory Etiquette**	Wash hands with soap and water (at least for 20 second)	Individually proceed to sink	154/197 (78.2%)	16/197 (8.1%)	27/197 (13.7%)
		Wipe hands discard appropriately	163/197 (91.1%)	10/197 (5.1%)	24/197 (12.2%)
		Apply hand disinfectant	152/197 (77.2%)	10/197 (5.1%)	35/197 (17.8%)
		Wear gloves	134/197 (68%)	30/197 (15.3%)	33/197 (16.8%)
	In case of coughs and sneezes	Mouth and nose covered	170/197 (86.3%)	6/197 (3%)	21/197 (10.7%)
		Used tissues thrown in the trash	169/197 (85.8%)	8/197 (4.1%)	20/197 (10.2%)
		hands washed immediately	149/197 (75.6%)	8/197 (4.1%)	40/197 (20.3%)
	Carry personal hand disinfectant		144/197 (73.1%)	19/197 (9.6%)	34/197 (17.3%)
	Avoid touching unnecessary surface or object		132/197 (67%)	14/197 (7.1%)	51/197 (25.9%)
	Do not touch the eyes, nose, or mouth		136/197 (69%)	20/197 (10.2%)	41/197 (20.8)
	Avoid sharing of electronic device, school materials, etc		135/197 (68.5%)	22/197 (11.2%)	40/197 (20.3%)
Mean			1638/2167 = 75.6	163/2167 = 7.5	366/2167 = 16.9
**Mandatory Disinfection**	Disinfectants permanently installed at entrance & exit		152/197 (77.2%)	10/197 (5.1%)	35/197 (17.8%)
	Each laboratory bench row contains:	Hand sanitizer	152/197 (77.2%)	22/197 (11.2%)	23/197 (11.7%)
		Hand soap	143/197 (72.6%)	32/197 (16.2%)	22/197 (11.2%)
		Surface disinfectant/ Antiseptic	138/197 (70.1%)	24/197 (12.2%)	35/197 (17.8%)
		Surface disinfectant	139/197 (70.1%)	22/197 (11.2%)	36/197 (18.3%)
		Trash/wastes bin	166/197 (84.3%)	7/197 (3.6%)	24/197 (12.2%)
		Paper towel	143/197 (72.6%)	21/197 (10.7%)	33/197 (16.8%)
	Spray disinfectants to surfaces regularly	Before and after the class	139/197 (70.1%)	21/197 (10.7%)	37/197 (18.9%)
		Whenever there is contamination	143/197 (72.6%)	12/197 (6.1%)	42/197 (21.3%)
	Individual disinfecting a objects handled in between use		125/197 (63.5%)	12/197 (6.1%)	60/197 (30.5%)
Mean			1440/1970 = 73.1	183/1970 = 9.3	347/1970 = 17.6
**Cleaning & Waste Disposal**	Practice proper laboratory waste segregation and disposal		151/197 (76.6%)	13/197 (6.6%)	33/197 (16.8%)
	Routine daily collection of generated wastes by janitors		144/197 (73.1%)	12/197 (6.1%)	41/197 (20.8%)
Mean			295/394 = 74.9	25/394 = 6.3	74/394 = 18.8
**Laboratory Procedure Prior To Leaving The Room**	disinfect the instruments, device, & working area		134/197 (68%)	22/197 (11.2%)	41/197 (20.8%)
	Discard the wastes and gloves properly		169/197 (85.8%)	6/197 (3%)	22/197 (11.2%)
	Wash hands with soap and water (individually)		159/197 (80.7%)	6/197 (3%)	32/197 (16.2%)
	Wipe hands dry & discard appropriately		156/197 (79.2%)	9/197 (4.6%)	32/197 (16.2%)
	Used lab gowns must be removed before leaving		124/197 (62.9%)	27/197 (13.7%)	46/197 (23.4%)
Mean			742/985 = 75.3	70/985 = 7.1	173/985 = 17.6
**Modified laboratory teaching strategies**	Student stays in assigned area and seat		197/197 (100%)	-----	-----
	Student disinfects the working area for evaluation		197/197 (100%)	-----	-----
	Student moves 2-meter away leaving the output to be checked by the evaluator		197/197 (100%)	-----	-----
	Student disinfects the working area after evaluation		197/197 (100%)	-----	-----
Mean			788/788 (100%)		
**Signs, posters, and engineering controls**	Availability of sticker showing seat assignment		197/197 (100%)	-----	-----
	Availability of sticker showing physical distancing		197/197 (100%)	-----	-----
	Availability of sticker showing hand washing technique		197/197 (100%)	-----	-----
	Availability of sticker showing signs & Symptoms		197/197 (100%)	-----	-----
	Availability of sticker showing stop spread		197/197 (100%)	-----	-----
Mean			985/985 (100%)		
Over all mean			7227/9259 = 78.1	686/9259 = 7.4	1346/9259 = 14.5

A checklist was designed to assess the compliance of each participant to the safety strategy. The interpretation of the checklist depends on marks: two marks indicates that the requirement is available and implemented, one mark indicates that the requirement is partially or sometimes implemented and zero marks indicates the item is neither available nor implemented.

The laboratory instructors monitored the practice of safety strategy among the study population and immediately corrected malpractice to ensure adherence to proper implementation.

### Detection of SARS-CoV-2 antibodies

Sterile disposable vacutainer tubes were used to collect 3 ml of blood from the antecubital vein under sterile conditions from each respondent, and the blood samples were allowed to clot at room temperature. The clotted samples were centrifuged at 5,000 rpm for 5 minutes to separate the sera; afterwards, the sera were frozen at -20°C until use.

The serological diagnosis of SARS-CoV-2 antibodies was performed at the end of the study period. The serum samples were tested for the presence of IgM+IgA and IgG by using an indirect immunoenzyme assay (Vircell- Spain). The tests were performed according to the instructions of the manufacturer. The participants who developed symptoms or signs of COVID-19 infection during the study period were referred to the authorized governmental center to undergo PCR tests for COVID-19.

### Data analysis

The assessment of compliance with the safety strategy was dependent on the checklist (as described above). The instructors regularly completed the checklist for each participant, and the final report represented the average of the responses. The response to each requirement and condition was calculated as a percentage by dividing the sum of the responses by the total number of participants. The chi-square test (SPSS version 22) was used to assess the statistical relationships between the requirements, conditions, study groups and seropositive results. The *P* value (0.05) was used for the justification of significance.

## Results

**Fig 1:**
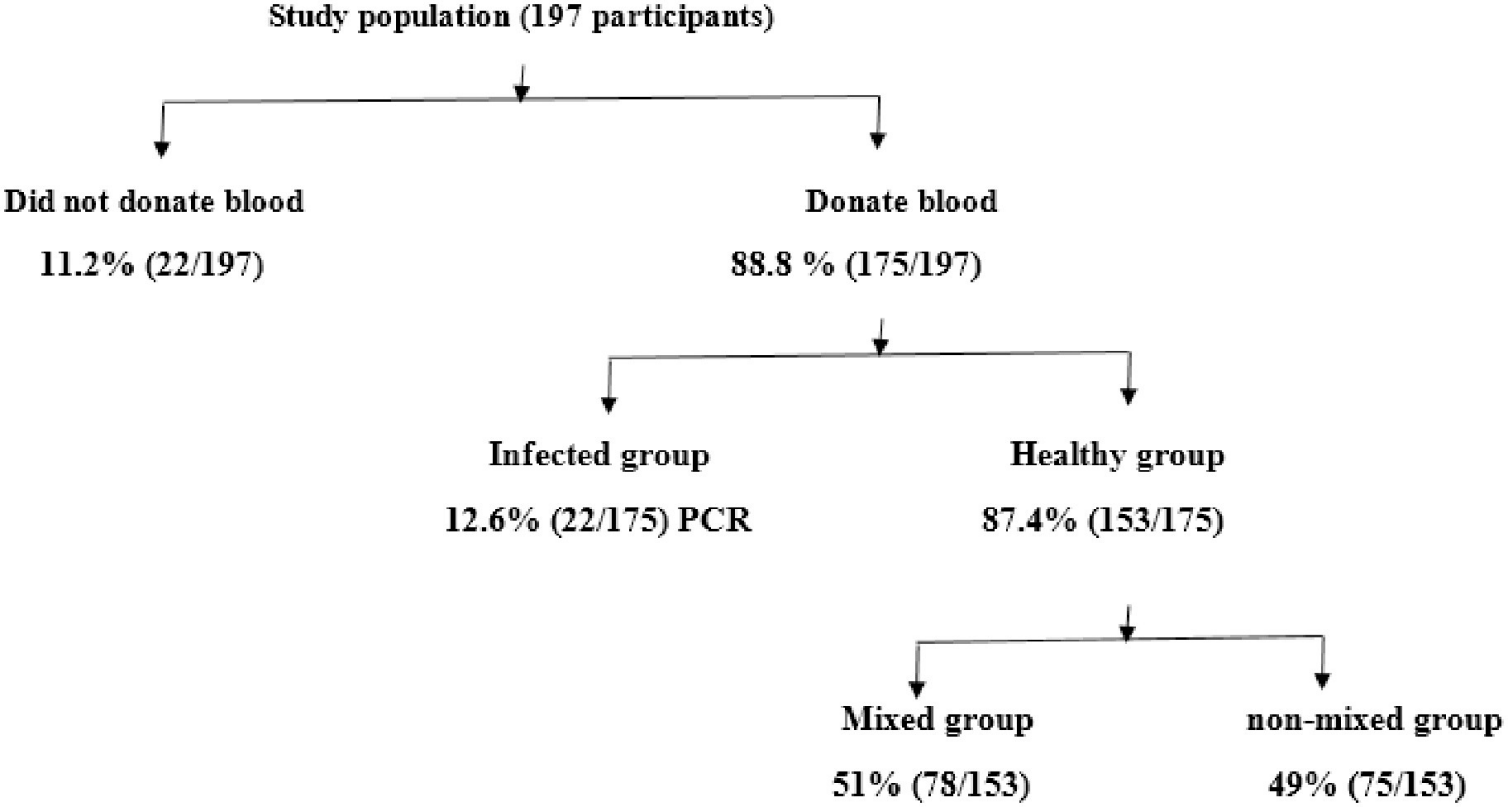
Distribution of study population.

A total of 197 students were enrolled at the beginning of the study; 11.2% (22/197) of the students completed the study but did not donate blood samples. The responses of this group to the safety protocol were considered, and they were ignored when calculating the serological results (Fig 1). A total of 25.4% (50/197) of the study population had chronic diseases.

Table 1 shows the level of response of participants toward the implemented safety strategy; moreover, a full response (100%) to the safety strategy was observed in the modified laboratory teaching strategies condition and in the signs, posters and engineering controls condition. The overall result showed that 78.1% of the respondents strictly followed the safety strategy, 14.5% of the individuals partially responded to the strategy and 7.4% of the individuals did not respond to the strategy.

Most of the respondents (75.6%, 75.3% and 74.9%) fulfilled the hand hygiene and respiratory etiquette condition, laboratory procedure prior to leaving the room condition, cleaning and waste disposal condition, respectively. The lowest compliance to the strategy was observed in the physical distancing conditions, in which 62.6% of the respondents obeyed these requirements.

**Fig 2:**
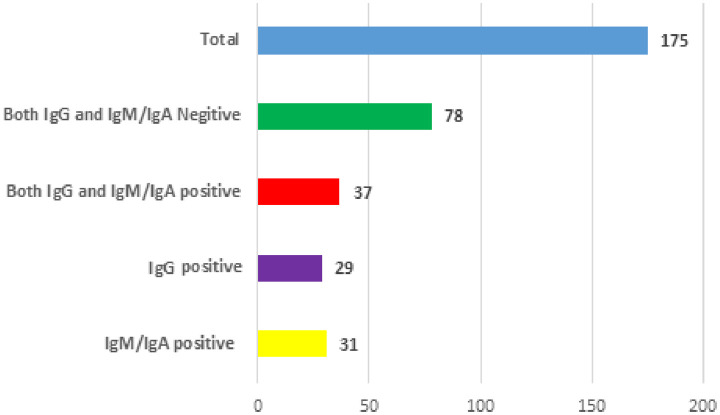
Serological results IgG and IgM/IgA antibodies.

A total of 12.6% (22/175) of the participants who completed the study and who donated blood had positive PCR results for COVID-19 infection during the study period (along with symptoms), and they represented the infected group; moreover, the remaining participants (87.4%, 153/175) did not show symptoms up to the end of the study, and they represented the healthy group. Furthermore, 51% (78/153) of the healthy group had mixed COVID-19 patients (mixed group), and 49% (75/153) of the individuals did not mix COVID-19 patients (non-mixed group) (Fig 1). The serological results showed that 38.9% (68/175) and 37.7% (66/175) of the study population had positive IgM+IgA and IgG antibodies against COVID-19, respectively; 51.5% (35/68) of the IgM+IgA candidates and 34.8% (23/66) of the IgG seropositive candidates had chronic diseases. Furthermore, IgM+IgA alone was detected in 45.6% (31/68) of the seropositive participants, whereas IgG alone was observed in 34.9% (29/66) of the seropositive participants (Fig 2).

The majority of IgM+IgA seropositive (73.5%, 50/68) and IgG seropositive (68.2%, 45/66) individuals belonged to the healthy group; out of these individuals, only 14% (7/50) of the seropositive IgM+IgA individuals and 37.8% (17/45) of the seropositive IgG individuals belonged to the non-mixed group (Tables 2 and 3).

**Table 2:** Serological results IgG and IgM+IgA of COVID-19 infection according to study groups.

Groups		IgM+IgA result				IgG result				total
		positive		Negative		positive		IgG Negative		
		frequency	%	frequency	%	frequency	%	frequency	%	
Infected group		18	81.8	4	18.2	21	95.5	1	4.5	22
Healthy group	Mixed group	43	55.1	35	44.9	28	35.9	50	64.1	78
	Non mixed group	7	9.3	68	90.7	17	22.7	58	77.3	75
Total		68	38.9	107	61.1	66	37.7	109	62.3	175

**Table 3:** Seropositive results IgG and IgM+IgA of COVID 19 infection among study groups.

Groups		IgM+IgA result		IgG result	
		frequency	%	frequency	%
Infected group		18	26.5	21	31.8
Healthy group	Mixed group	43	63.2	28	42.4
	Non mixed group	7	10.3	17	25.8
Total		68	100	66	100

**Fig 3:**
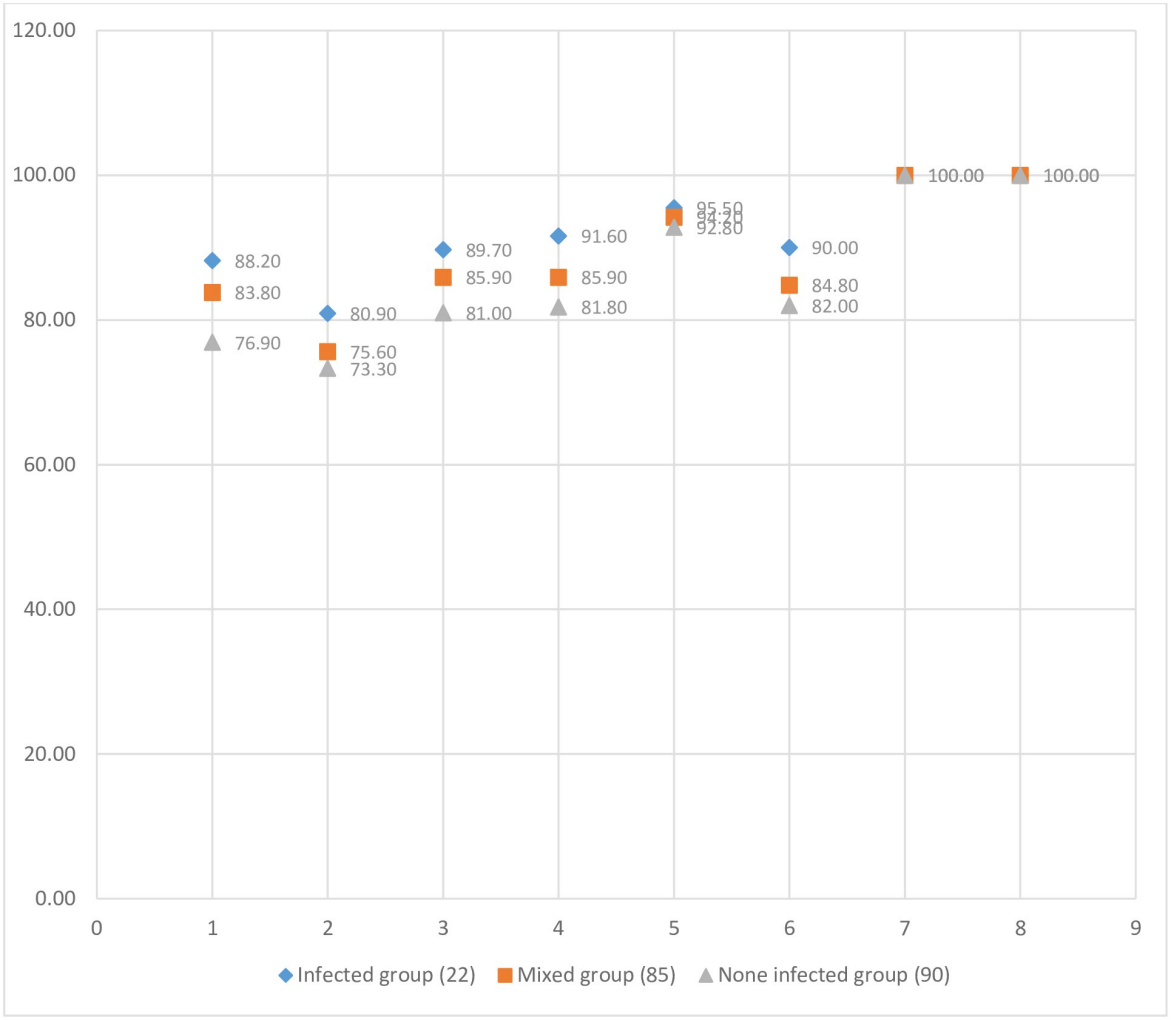
The response of the three groups; symptomatic group, mixed group and non-mixed group to the safety strategy. Footnote: the number in brackets represent the number of individuals per group; 1. Laboratory procedure upon entry 2. Physical distancing 3. Hand hygiene and respiratory etiquette 4. Mandatory disinfection 5. Cleaning & waste disposal 6. Laboratory procedure prior to leaving room 7. Modified suitable laboratory teaching strategies 8. Signs, posters, and engineering controls.

Fig 3 shows the response of the three groups (infected group, mixed group and non-mixed group) to the safety strategy. The highest compliance to the safety strategy was observed among the infected group, followed by a mixed group. The infected group showed a response to the safety protocol between 80.09% for physical distancing conditions and 95.5% for cleaning and waste disposal conditions.

The statistical analysis showed a significant relationship between seropositive IgM+IgA and chronic disease (*P* = 0.000). The physical distancing condition was the only condition that displayed a significant relationship with seropositive IgM+IgA (*P* = 0.045). Although numerous significant relationships were observed between the seropositive IgM+IgA and several requirements, it had a significant relationship with the daily assessment of temperature status (*P* = 0.003), entering the door individually (*P* = 0.035), individually washing hands with soap and water (*P* = 0.020) and covering the mouth and nose in cases of coughs and sneezes (*P* = 0.036). When considering the study groups, the infected group (*P* = 0.000) and the mixed group (*P* = 0.000) demonstrated a significant relationship with seropositive IgM+IgA. Seropositive IgG did not show any relationship to conditions or requirements.

## Discussion

The increase in COVID-19 (which is caused by SARS-CoV-2), along with its continued waves and the emergence of new variants of concern (VOC), poses a significant health threat and affects medical professionals in their training, as well as in their academic and professional development [20, 21]. The best manner of prevention is to avoid being exposed to the virus [8], particularly the last emerged variants that showed partial resistance to the available vaccines [22]. Therefore, the implementation and assessment of the safety strategy plays a vital role determining the community’s readiness to deal with the changes and provides useful insights to treat poor practices [23].

Many studies have determined the knowledge, perceptions, awareness and preventive practices toward COVID-19 among different groups [3, 7, 23–29]. All of these studies were conducted over a short period of time, and these studies involved cross-sectional surveys, whereas our study was an interventional descriptive study that traced the preventive practice level toward COVID-19 over a time period of 4 months. This study built a model comprising eight conditions, with each condition involving related factors instead of separate assessments of each factor. In alignment with this concept, Kebede et al., 2020 stated that the adaptation of preventative practice as packages is essential to save the community from the risk of contracting the infection [25].

The present study showed that most of the participants (78.1%) completed the protocol. A 100% response to the safety strategy was observed in the modified laboratory teaching strategies condition and in the signs, posters and engineering controls condition, whereas 75.6%, 75.3% and 74.9% of the participants fulfilled the hand hygiene and respiratory etiquette condition, laboratory procedure prior to leaving the room condition, cleaning and waste disposal condition, respectively. The lowest obedience to the safety strategy was observed in the physical distancing condition, in which 14.6% of the study population did not apply and 22.7% of the population did partially apply. Physical distancing conditions and several requirements, such as daily assessments of temperature, entering the door individually, washing hands with soap and water individually and covering mouth and nose in cases of coughs and sneezes, exhibited a significant relationship with seropositive IgM+IgA. These findings were relatively comparable with global published data, which showed that the highest response was toward wearing masks (82.7% to 100%), followed by washing hands (77.3% to 100%). Similar to our findings, a lower response was reported against physical distancing and avoiding handshaking by several authors [3, 7, 25, 26, 29]. However, Mishra et al., 2020 performed a study among medical students to assess awareness of COVID-19, and they reported that 91.1% and 100% of medical students had an awareness of wearing masks and washing hands, respectively [26].

Our findings were relatively similar to those reported among the Saudi Arabian population, where 84%, 75%, 82%, 79% and 76% of the study population knew and practiced washing hands, sneezing or coughing into the arm/elbow, avoiding shaking hands, keeping a safe distance and avoiding touching one’s face, respectively. Our study showed better performance, in which only 7.4% of the participants did not respond to the safety strategy, whereas 16.2% to 25% of the Saudi population had the knowledge and practiced the knowledge [29]. These variations may be due to differences in the study population, duration and type of the study. Generally, the practices toward COVID-19 were not at the required level in contrast to the contagious nature of the virus [29].

Our study showed that the majority of the participants (153, 87.4%) completed the study without showing symptoms, and only 22 (12.6%) respondents developed COVID-19 symptoms and had positive PCR results for COVID-19. These results may indicate the success of the safety protocol in reducing SARS-CoV-2 transmission. The serodiagnosis demonstrated that 68 (38.9%) and 66 (37.7%) of the participants had positive IgM+IgA and IgG of COVID-19, respectively. Globally, the seroprevalence of SARS-CoV-2 was low in the general population and varied greatly between different communities (from 0.6% to 59%). The median seroprevalence varied according to the Global Burden of Disease area from 0.6% in East Asia, Oceania and Southeast Asia to 19.5% in Sub-Saharan Africa [30]. The seropositivity in our study indicated a relatively high exposure to SARS-CoV-2, and these results could be partially due to non-compliance and poor practice with the protocol (14.5% of the participants partially responded to the protocol, and 7.4% of the participants did not respond) or to exposure outside of the university campus. However, Mohapatra et al., 2021 concluded that adherence to the COVID‐19 safety policy to the desired levels by students in colleges and schools is truly challenging [22]. The physical distancing condition demonstrated a significant relationship with seropositive IgM-IgA, and this finding was consistent with the most frequent mode of transmission in Saudi Arabia, wherein 95% of the cases have been the result of social gatherings [4]. Another report showed that 54 medical students were COVID‐19 positive as a result of social gatherings [22]. Our study demonstrated the role of gathering in the transmission of disease, as reflected by the statistical relationship between entering the door individually, individually washing hands with soap and water, mixed group and seropositive IgM-IgA. Similarly, Ramage-Morin and Polsky 2020 [31] and Bhusare et al., 2020 [1] reported that social gathering is the main source of transmission. Furthermore, our study determined that the majority of respondents who developed SARS-CoV-2 antibodies did not develop symptoms, which may be due to abortive infections or asymptomatic infections. This finding was consistent with the results of Naiyar et al., 2021, who determined the seroprevalence of SARS-CoV-2 in a medical institution and stated that most (68.42%) of the technical staff and administrators who had positive IgG were asymptomatic [32]. Nevertheless, the asymptomatic individual can act as the source of the disease (silent transmission), as asymptomatic infections have the same infectivity as symptomatic infections [9]. Several reports have shown that most COVID-19 transmissions occur from presymptomatic or asymptomatic patients [33]. Interestingly, in our study, the presence of asymptomatic patients did not lead to the overspread and development of the disease, as indicated by a limited number of the participants who had positive PCR results for COVID-19 during the study. These findings may reflect the role of the protocol in controlling and reducing the exposure time, which leads to sub-infective doses.

The present study showed that the infected group had the highest compliance with the safety protocol, followed by the mixed group. These results may appear to be contradictory, but they could be interpreted by the protocol itself, in which each candidate who showed symptoms or had mixed COVID-19 patients was prohibited from attending the classes until they showed negative PCR results. Subsequently, special care and follow-up concerning the implementation of the safety protocol were provided to candidates while attending the classes.

### Limitations of the study

The present study had two main limitations. First, exposure to COVID-19 outside of the university campus (public transport, markets, etc.) may be the main source of infection, and our study did not include tools to measure the level of response to the safety strategy outside of the university. Second, the asymptomatic patients were identified at the end of the study when the serological test was performed, and it will be more effective if the serological test is performed based on a fixed interval, which may help in identifying asymptomatic patients at a suitable time and in breaking down one of the major routes of transmission.

## Conclusions

A coordinated, standardized safety strategy combined with follow-up and laboratory diagnosis can be an effective tool in measuring the effectiveness of a safety strategy and in reducing the risk of exposure to COVID-19 infection, thus cutting off the route of transmission and enabling suspended activities, particularly in countries where there is a shortage of vaccine supply or a resistance to vaccination.
